# Working Memory Constrains Long-Term Memory in Children and Adults: Memory of Objects and Bindings

**DOI:** 10.3390/jintelligence11050094

**Published:** 2023-05-15

**Authors:** Alicia Forsberg, Dominic Guitard, Eryn J. Adams, Duangporn Pattanakul, Nelson Cowan

**Affiliations:** 1 Department of Psychology, University of Sheffield, Vicar Ln, Sheffield S1 2LT, UK; 2 School of Psychology, Cardiff University, Tower Building, 70 Park Place, Cardiff CF10 3AT, UK; 3 Psychology Building, The University of New Orleans, 2000 Lakeshore Drive, New Orleans, LA 70148, USA; 4 Department of Psychology, University of Tennessee, Austin Peay Hall, Knoxville, TN 37996, USA; 5 Department of Psychological Sciences, University of Missouri, McAlester Hall, Columbia, MO 65211, USA

**Keywords:** working memory, child development, long-term memory, information transfer, memory binding

## Abstract

We explored how individual and age-related differences in working memory (WM) capacity affected subsequent long-term memory (LTM) retrieval. Unlike past studies, we tested WM and LTM not only for items, but also for item–color bindings. Our sample included 82 elementary school children and 42 young adults. The participants performed a WM task with images of unique everyday items presented sequentially at varying set sizes in different colors. Later, we tested LTM for items and item–color bindings from the WM task. The WM load during encoding constrained LTM, and participants with a higher WM capacity retrieved more items in the LTM test. Even when accounting for young children’s poor item memory by considering only the items that they did remember, they exhibited an exacerbated difficulty with remembering item–color bindings in WM. Their LTM binding performance, however, as a proportion of remembered objects, was comparable to that of older children and adults. The WM binding performance was better during sub-span encoding loads, but with no clear transfer of this benefit to LTM. Overall, LTM item memory performance was constrained by individual and age-related WM limitations, but with mixed consequences for binding. We discuss the theoretical, practical, and developmental implications of this WM-to-LTM bottleneck.

## 1. Introduction

To navigate our daily cognitive challenges, we rely on both working memory (WM) and long-term memory (LTM). WM^1^ is a system that holds a limited amount of information in mind for use in ongoing cognitive tasks, and this system is often described as a mental workspace (Adam et al. 2017; Cowan 2001, 2017; Logie et al. 2020). Long-term memory (LTM) refers to the ability to commit new information—such as words, facts, or concepts—to a long-term memory store for retrieval minutes, days, or even decades later. While our working memory capacity appears highly limited (e.g., Ngiam et al. 2022), we seem able to store effectively unlimited amounts of information in our LTM (e.g., Brady et al. 2008; Standing 1973). 

Both these memory systems play crucial roles in childhood cognitive development. While working memory appears essential to solving new problems and learning new concepts (Cowan 2022), children’s gradual accumulation of long-term knowledge helps them navigate their daily lives safely and progress in formal education (Alloway and Alloway 2010; Gathercole et al. 2004). Typically, as children develop, their memory performance improves (see Forsberg et al. 2022c). However, the developmental trajectory of LTM ability is not necessarily straightforward, as children’s memory abilities appear very good in some ways and poor in others (Miller 2014). For example, young children may be considered less reliable court witnesses than adults due to their poor memory abilities (e.g., Knutsson and Allwood 2014). However, under some circumstances, their memory accuracy can be similar to that of adults (e.g., Jack et al. 2014). On the other hand, visual working memory capacity typically shows consistent development throughout school years (e.g., Cowan 2016; Cowan et al. 2011; Gathercole et al. 2004). The importance of WM in children’s ability to navigate both their everyday lives and formal education is highlighted in a large body of correlational research which suggests that individual differences in WM capacity quite reliably predict subsequent educational attainment (Allen et al. 2021; Batool and Saeed 2019; Gathercole et al. 2004). This relationship may be driven by numerous factors. For example, WM capacity and educational attainment may both be correlated with fluid intelligence, which is closely linked to WM capacity (Conway et al. 2003; Rey-Mermet et al. 2019; Unsworth et al. 2014) and may also support educational attainment more broadly.

Recently, it has been suggested that correlations between higher WM capacity and greater educational attainment might be partially explained via the increased transfer of information to LTM in individuals with a higher WM capacity (Forsberg et al. 2021a). In general, across one’s lifespan, LTM retrieval is far from perfect as we are often exposed to information that is rapidly forgotten. As an example, neither children nor adults are likely to remember all the information that was presented during a school lesson. Recent work using a variety of paradigms has suggested that time-related encoding limitations or WM limitations may result in a limited funnel through which to learn new things (Bartsch et al. 2019; Čepukaitytė et al. 2023; Cotton and Ricker 2021; Forsberg et al. 2021a, 2021b, 2022a, 2022b; Fukuda and Vogel 2019; Loaiza et al. 2020; Rivera-Lares et al. 2022). Specifically, Forsberg and colleagues found that WM capacity limitations constrained the number of items children (Forsberg et al. 2022a), young adults (Forsberg et al. 2021a), and older adults (Forsberg et al. 2022b) recognized in a later LTM test. In these studies, LTM performance was better for items presented as part of a lower WM load (e.g., items presented within an array of two items were better remembered than items presented in an array of six items, even when the encoding time was adjusted according to the number of items). Moreover, they found that individual differences in WM capacity within age groups explained individual differences in LTM performance. Finally, this past research also suggested that children’s WM limitations may explain their LTM deficits since the ratio of the information that was transferred into LTM—out of what participants could hold in WM—appeared relatively consistent across age groups. 

In the current paper, we expanded these significant findings using a task that required memory for bound information (i.e., item–color bindings) presented sequentially (i.e., one item at a time). The rationale for these expansions is as follows. First, Forsberg et al. only measured item memory, but some argue that the maintenance of WM bindings characterizes the limits of WM capacity (Oberauer 2005, 2019; Schneegans and Bays 2019). Recent research suggests that visual WM performance improves across childhood due to increases in both the number of objects and the amount of feature detail that can be held in WM (Forsberg et al. 2022d). Memory for color–location bindings has been demonstrated in infants as young as 7.5 months (Oakes et al. 2006), and WM for various types of feature bindings (e.g., color–location, object–background, verbal–spatial, or visual–auditory) appears better in adults than in school-aged children (e.g., Cowan et al. 2006a, 2006b; Darling et al. 2014; Wang et al. 2015). In this study, we explored age differences in the ability to retain memory for bindings in both WM and LTM. 

Second, Forsberg et al. presented all items simultaneously. Thus, the effect of WM set size on subsequent LTM retrieval may be caused by a shared perceptual encoding bottleneck rather than a WM capacity limitation per se, because their higher set size arrays were inherently more visually complex given the simultaneous presentation of items. Providing some comfort, Cowan et al. (2011) found comparable performance levels for sequential lists and simultaneous arrays of colored squares in a change detection task, suggesting that WM capacity was the limiting factor. Nevertheless, if the core findings of Forsberg et al. (2022a) do not replicate with the sequential presentation, it would suggest that the perceptual load, rather than WM, limited LTM in these previous studies. Confirming whether WM capacity constrains LTM with sequential presentation also has practical importance for educational settings, in which new information can be encountered both sequentially (e.g., in a lesson, video clip, or book) or simultaneously (e.g., on wall displays and whiteboards; Atkinson et al. 2019). Combined, these research questions will improve our understanding of how WM capacity changes with age, and how WM limitations restrict subsequent LTM retrieval.

## 2. Method

### 2.1. Participants

#### 2.1.1. Sample Size Determination

We based our target sample size on the study conducted by Forsberg et al. (2022a), which included 40 participants per age group. Our final participant numbers were determined by the availability of the participants. While Forsberg et al. included four age groups, we only included three (i.e., we did not include 3rd and 4th graders) as the greatest developmental effects should be observed at the more extreme ranges of the age spectrum. Specifically, the participants included children in first and second grade, fifth through seventh grade, and young adults (see Section 2.1.4).

#### 2.1.2. Recruitment

We recruited child participants via local schools and social media. We also invited local college students to participate either for course credit or financial reimbursement. Participants received USD 15 for completing the study, and additional compensation if the session exceeded 45 min. All participants gave their informed consent before participating, and the study was approved by the local ethics (IRB) committee.

#### 2.1.3. Exclusions 

We excluded three participants in the youngest age group (1st and 2nd graders) and one in the older child group (5th through 7th graders) for near chance-level performance in the easiest condition, defined as item memory accuracy of less than 55% at set size two in the WM task. This exclusion criterion was similar to Forsberg et al. (2022a) and was implemented to filter out participants who were responding randomly, likely due to failure to understand or engage with the task instructions.

#### 2.1.4. Final Sample

The final sample included a total of 124 participants from 3 different age groups: 1st and 2nd graders (*N* = 43, *M* = 6.5, *SD* = 0.59 years, 44.2% male, 53.5% female, and 2.3% prefer not to say), 5th through 7th graders (*N* = 39, *M* = 11.7, *SD* = 0.77 years, 59.0% male, and 41.0% female), and young adults (*N* = 42, *M* = 20.4, *SD* = 2.3 years, 38.1% male, 59.5% female, and 2.4% other). 

#### 2.1.5. Online Study Administration

We collected data online, using an online communication software (Zoom), and the study was programmed in PsyToolkit (Stoet 2010, 2017). Using this approach, the experimenter was able to explain the study instructions, answer questions, and provide encouragement. While the participant was completing the experimental task, the experimenter muted their webcam audio and turned off the video, except to provide a minimum of three positive reinforcement phrases (e.g., “Hang in there!”). If the participant appeared to disengage from the task, the experimenter provided additional encouragement. 

### 2.2. General Study Design

Each participant completed three different tasks in a fixed order: (1) a WM probe-recognition task, (2) four trials of an unrelated visuospatial memory distraction task to remove any remnants of the stimulus representations from WM, and (3) an LTM probe-recognition test, assessing memory for the items previously studied in the WM task. 

### 2.3. WM Task

**Figure 1:**
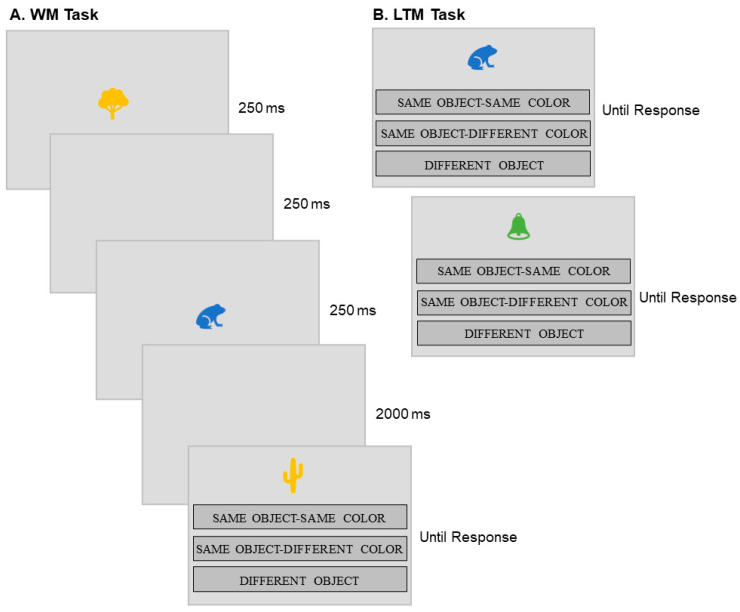
Outline of some typical trials. **Panel A**, working memory (WM) task trial, at set size 2. **Panel B**, two trials in the long-term memory (LTM) task. The memory array set size in the WM task varied between 2, 3, and 4 items. Participants responded using one of three response options “same object–same color”, “same object–different color”, or “different object”.

First, the participants studied a total of 336 unique memory items in a WM task, presented sequentially in 1 of 6 colors (blue, brown, green, indigo, orange, or red), at varying set sizes (2, 3, or 4 items; see Figure 1). The memory items were selected from the Microsoft Office Icons and consisted of easily recognizable images, including different types of animals, fruits, furniture, etc. Each item was presented for 250 ms, followed by a blank screen (250 ms) and then the next item. Each participant completed a total of 120 WM trials (54 trials at set size 2, 36 trials at set size 3, and 30 trials at set size 4), always with 1 probe per trial. Within these, in one-third of the trials, the probe item was identical to one of the array items (Same Object, Same Color). In another third of the trials, the probe item was identical to one of the items in the array but presented in a different color (Same Object, Different Color). Out of these, half of the objects were presented in the color of a different object from the original array, and the second half of the objects were presented in a color that was not included in the original array. In the final third of the trials, the probe item was a different object (Different Object). Half of these different objects were presented in a color from the array, and the other half were presented in a color which was not included in the original array. 

The order of trials and items was randomized for each participant. Memory was measured in the WM task by asking participants to respond to a single probe item presented in one of the six colors used in the study. They responded by clicking on one of the following options on the screen: ‘SAME OBJECT–SAME COLOR’, ‘SAME OBJECT–DIFFERENT COLOR’, or ‘DIFFERENT OBJECT’. Each trial started with a 250 ms central fixation cross, followed by the memory array, which consisted of 2, 3, or 4 items, presented sequentially for 250 ms per item, with a 250 ms blank screen in between. After the final item in the sequence, there was a 2000 ms delay before the probe item and response options were presented (see Figure 1). 

### 2.4. Distraction Task

The participants completed four trials for a brief, unrelated visual memory task. During this task, aliens appeared in a three-by-three grid, and the participants were asked to click on the grid locations in which the aliens had been presented. Two of the trials presented a set of four aliens, and the other two trials had a set of five aliens.

### 2.5. LTM Task

In this final phase, we tested the participants’ memory for the items they had previously studied in the WM task. On each trial, the participants saw a single probe item. The participants responded using the same response options as in the WM task (‘SAME OBJECT–SAME COLOR’, ‘SAME OBJECT–DIFFERENT COLOR’, or ‘DIFFERENT OBJECT’). Each participant responded to a total of 144 items in the LTM test (48 new items, 32 items from set size 2, 32 items from set size 3, and 32 items from set size 4). Out of the 32 studied items at each set size, 16 were presented in a different color (i.e., Same Object, Different Color trials). In 50% of these trials, the different color was not part of the original array, and in the remaining 50%, it was one of the colors from the original array. The remaining 16 trials from each set size were Same Object, Same Color trials. In half of these trials, the LTM probe item had also been used as a probe item in the WM task. Thus, the LTM test consisted of a mix of items that were and were not tested in the WM test, spread evenly across the three different set sizes.

### 2.6. Analytical Approach

In this section, first, we discuss the Bayesian method of statistical inference that we used. Then, we note special considerations in the analysis of the item and binding information, respectively. 

#### 2.6.1. Bayes Factors

In the analyses reported below, we used a Bayesian model comparison approach, and a nomenclature in which BF_10_ refers to the Bayes Factor for the presence of an effect and BF_01_ refers to an absence of an effect, such that BF_01_ = 1/BF_10_. Models were compared using the ‘generalTestBF’ function in the BayesFactor package for R (Morey et al. 2015). Individual participants were included as random effects in all models. All data and analysis codes are available via the Open Science Framework. (https://osf.io/9zvrm/, accessed on 20 April 2023). 

#### 2.6.2. Item Memory 

For our item memory measure, responses were scored as correct when participants used one of the two ‘Same Object’ response options, regardless of whether they were correct about the items’ color. If the object was different, the response was scored as correct only if the participant used the ‘Different Object’ response option. Using these values, we estimated the proportion of items from a given WM set that were remembered during (1) WM testing; *p*(WM), and (2) LTM testing; *p*(LTM), for each participant. Specifically, our *p*(WM) estimates were obtained by determining the rate at which the participants correctly detected the studied items (i.e., the hit rate, *h*) and the rate at which they incorrectly responded that new items were old (i.e., the false alarm rate, *f*). To estimate *p*(WM), we applied an approach designed by Pashler (1988) adjusted to a single-probe test scenario by Cowan et al. (2013). The approach relies on the following assumptions: when participants remember the probed item, they should respond correctly; when participants do not remember the item, they should guess that the item is new with a certain rate (*g*). Thus, the rate of the correct detection of old items, *h*, equals the probability that the probe item is remembered plus the probability that it was not remembered but that a correct “old” guess *g* is made:

*h = p*(WM) + [1 − *p*(WM)](*g*) (1)

For a new item, performance depends on the guessing rate, such that an incorrect response (*f*) will be made at the rate *f* = *g*. By combining these formulas, it can be shown that:

(2) $$p(WM)={\left(\frac{h-f}{1-f}\right)}_{}$$

One can also use these results to estimate *k*, the number of items in WM, under the assumption that *p*(WM) = *k*/*S*, where *S* is the set size, so that *p*(WM)(*S*) = *k*.

We also used a similar approach to estimate the proportion of items in LTM. Here, hits (*hl*) are the rate of correctly detected old items, and false alarms (*fl)* are the rate at which the participants incorrectly responded that a novel item was old:

(3) $$p(LTM)={\left(\frac{hl-fl}{1-fl}\right)}_{}$$

#### 2.6.3. Theoretically Implausible Values

When using this approach, if the false alarm rate exceeds the hit rate, *p*(WM) or *p*(LTM) becomes a negative value. This makes little theoretical sense and is likely to be the result of poor memory combined with unlucky guessing. In the analyses below, we deal with instances of such negative values by adjusting them to theoretically plausible values. Specifically, *p*(WM) values equivalent to a WM capacity of less than 1 were adjusted to equal 1, and *p*(LTM) values < 0 were adjusted to 0. 

#### 2.6.4. Binding Memory

Our task was designed to examine multiple possible influences on memory for the binding between an ordinary object and an auxiliary feature, its color, as well as memory of the item itself. In our analyses, we assessed these influences in a contingent manner. There were two trial types indicative of how often binding occurred, given that the object was remembered: (1) Same Object, Same Color, and (2) Same Object, Different Color (i.e., the object was the same but presented in a different color). These resulted in two correct-binding response types, 1a, participant correctly indicates object and color same; 2a, participant correctly indicates that the object is the same and that the color has switched, and two incorrect-binding response types, 1b, participant incorrectly says that the color has switched; 2b, participant incorrectly says that nothing has changed when, in fact, the color has changed. To assess binding memory performance, we compared the relative differences between these scenarios. To assess age differences in binding memory, we distinguished between three possible outcomes in these trials. First, we split items based on whether the object was correctly identified as studied (vs. forgotten). Then, in the trials in which the object was (correctly) identified as studied, there are two possible outcomes: (1) *Binding Error* (i.e., they remember the item, but are wrong regarding its color) or (2) *Binding Success* (they remember the item and were right about its color). We report age and set size differences in these parameters below. 

## 3. Results

We begin with the reporting of item memory, followed by binding memory. After examining WM-LTM correlations, we finally include a brief description of distraction task performance.

### 3.1. Item Memory

#### 3.1.1. Item Working Memory

**Figure 2:**
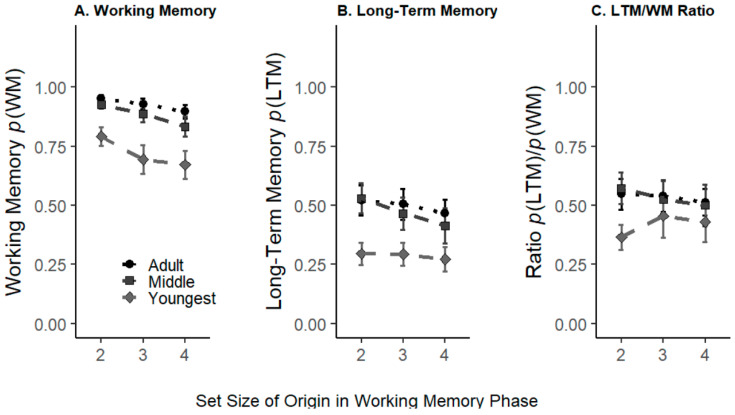
Memory accuracy by WM set size and age group. **Panel A**, *p*(WM) (i.e., the estimated probability that items were held in WM); **Panel B**, *p*(LTM) (i.e., the estimated probability that items were held in LTM); **Panel C**, the *p*(LTM)/*p*(WM) ratio. Black circles show adult data, squares data from the 5 to 7th graders, and diamonds represent data from the 1st to 2nd graders. Error bars represent 95% confidence intervals.

First, we tested how set size affected WM item accuracy. Similar to Forsberg et al. (2022a), we found that memory performance decreased as set size increased (BF_10_ = 1.20 × 10^11^), and that performance was better in older compared to younger participants (BF_10_ = 5.21 × 10^12^). Finally, we observed weak evidence against an age group by set size interaction (BF_01_ = 3.25, see Figure 2, Panel A). See Table 1 for all mean values by set size and age group. 

#### 3.1.2. Item Long-Term Memory 

Next, we tested whether LTM performance (*p*(LTM)) varied as a function of age group and WM set size. Performance was better for lower-set size items (BF_10_ = 3.90 × 10^3^) and in older compared to younger participants (BF_10_ = 1.87 × 10^5^), thus replicating the core findings of Forsberg et al. (2022a). However, evidence regarding an age group by set size interaction was inconclusive (BF_01_ = 1.09, see Figure 2, Panel B). 

False alarms (responding “studied” to novel items in the LTM test) of course did not differ by set size but were included in the calculation of *p*(LTM). There was a developmental change in bias, which was well known from previous work (e.g., Cowan et al. 2006b), in which younger children are more likely to believe that if they do not remember an item, then it certainly did not occur. As a consequence of this bias, younger children produced fewer such false alarms (1st and 2nd graders, M = .13, SD = .16; 5th through 7th graders, M = .29, SD = .16; young adults, M = .32, SD = .18) despite the developmental increases in *p*(LTM).

### 3.2. LTM/WM Ratio: Analysis of the Rate of Transfer of Information between WM and LTM 

Next, we assessed how many of the items encoded into WM could be remembered in the LTM test (i.e., the LTM/WM ratio shown in Figure 2, Panel C). This ratio is formed by dividing *p*(LTM) by *p*(WM) for each participant and set size. While the LTM/WM ratio appeared to vary across age groups (BF_10_ = 7.33), it did not seem to differ by set size (BF_01_ = 4.12). Finally, we observed weak, inconclusive evidence for an age group × set size interaction (BF_10_ = 1.13; see Figure 2). To follow up on this inconclusive interaction, we explored age effects at each set size individually. While the evidence for an age difference in the LTM/WM ratio was strong at set size 2 (BF_10_ = 4.24 × 10^3^), we observed some evidence against age differences at set sizes 3 (BF_01_ = 4.06) and 4 (BF_01_ = 4.03), suggesting that older participants benefitted from a more efficient ratio transfer at the very lowest set size (see Figure 2, Panel C). As in previous work, this outcome can occur because set size 2 may be close to the maximum WM capacity for the youngest group, but below WM capacity in the older groups, affording these older groups a more comfortable situation for the use of attention, compared to the youngest group. 

### 3.3. Binding Memory

#### 3.3.1. Correctly Identified Objects in Binding Trials 

First, we report the rates at which studied items were correctly identified as such between age groups and set sizes. Note that this measure does not account for the rate at which novel items were correctly identified as novel; such biases are accounted for in the *p*(LTM) measure reported above. Here, instead, we are simply interested in comparing the rates at which studied objects were correctly identified as such. These include trials with no change and trials with a changed color. The reason is to examine the age difference in the proportion of trials that will be eligible to use for an examination of binding differences, because the latter examination must exclude trials in which the object was not correctly identified. 

In the WM task, the rate of correct identification of studied items differed between age groups (BF_10_ = 2.27 × 10^10^) and set size (BF_10_ = 6.14 × 10^10^), with evidence against an interaction (BF_01_ = 71.68). Overall, the youngest children (*M* = .79, *SD* = .40) appeared to correctly identify fewer items as ‘studied’ than older children (*M* = .92, *SD* = .28) and adults (*M* = .95, *SD* = .23).

In the LTM task, this rate also differed between age groups (BF_10_ = 1.52 × 10^11^) and set size (BF_10_ = 2.33 × 10^2^), with some weak evidence against an interaction (BF_01_ = 3.78), as the youngest children appeared to correctly identify fewer items as studied compared to the older children and adults (younger children: *M* = .30, *SD* = .46, older children: *M* = .56, *SD* = .50, and adults: *M* = .63, *SD* = .48).

#### 3.3.2. The Rate of Binding Errors within Remembered Objects

Then, we explored the binding errors rates. For these analyses, we only included trials in which items were correctly identified as studied. Moreover, we did not include the *Same Object, Different Color* trials in which the item was presented in a novel color (i.e., a color that was not part of the memory array), since successful binding performance in this scenario might be achieved simply by noticing that the color is novel (rather than correctly remembering the item–color binding, per se). In these trials, we calculated the *Binding Error* rates as the rate at which participants remembered the object but were wrong regarding its color. For example, for a *Same Object, Same Color* trial, a binding error occurred when they responded, “Same Object, Different Color”, i.e., they incorrectly indicated a color change. 

**Figure 3:**
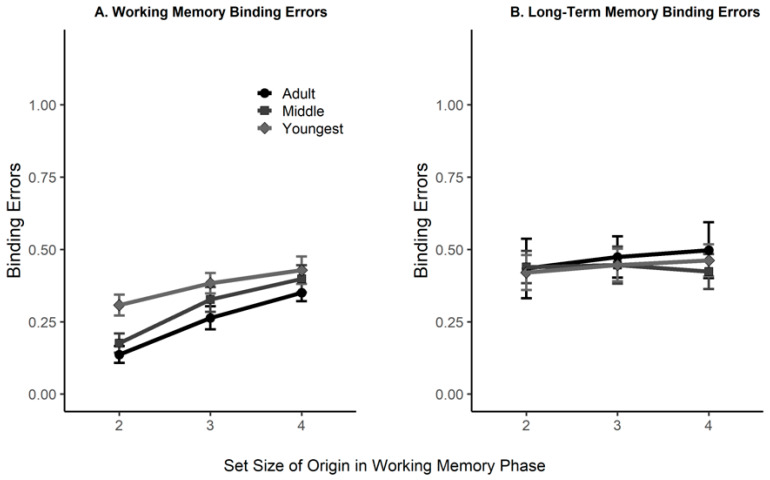
Binding memory accuracy rates by WM set size and age group, in trials which tested binding memory (Same Object, Same Color and Same Object, Different Color), in which items were not forgotten. **Panel A**, working memory binding error rates. **Panel B**, long-term memory binding error rates. Black circles show adult data, squares data from the 5 to 7th graders, and diamonds represent data from the 1st to 2nd graders. Error bars represent 95% confidence intervals.

Working Memory. In the WM task, the binding error rate appeared to differ between age groups (BF_10_ = 3.47 × 10^6^) and set sizes (BF_10_ = 7.95 × 10^28^), such that younger children committed more binding errors, and overall, error rates were greater for the higher set sizes. Moreover, there was inconclusive evidence for an interaction (BF_10_ = 2.16), potentially reflecting that the set size effect appeared less pronounced in the youngest children (see Figure 3, Panel A). Overall, younger children appeared to struggle more to remember bindings in WM in this analysis, which accounted for their higher rate of forgotten items.

Long-Term Memory. For the LTM binding analyses, we excluded trials in which the item was used as a WM probe item, as it might inflate the hit rates (i.e., correct identification of an item and its color as originally presented together). Given that the number of trials which were eligible to be included in this analysis depended on item memory accuracy (because trials in which a participant forgot the item were not informative regarding their binding memory), in some cases, data for a specific participant at a specific set size were sparse. To exclude potentially unreliable estimates, we excluded cases in which an estimate relied on fewer than 5 trials, which resulted in the exclusion of 71 out of 361 instances. This resulted in 67 eligible instances in the youngest age group, 103 in the middle group, and 120 in the adults. We found that the binding error rate did not differ between age groups (BF_01_ = 15.37), evidence regarding an effect of set size was inconclusive (BF_01_ = 2.73), and we observed some evidence against an interaction (BF_01_ = 9.12). Similar results were found when including all available instances (i.e., even those that consisted of data from fewer than five trials). Specifically, in that analysis, the binding error rates did not differ between age groups (BF_01_ = 8.89), with weak evidence against an effect of set size (BF_01_ = 3.13), and we observed evidence against an interaction (BF_01_ = 16.45).

Thus, when taking into account age differences in object memory, the rate of LTM binding errors appeared consistent across age groups (see Figure 3, Panel B). Remember, however, that this rate is based on far fewer eligible trials for younger children, and thus reflects an increase over age in the *number* of trials with a successful binding performance. 

Table 2 offers a more complete summary of the responses that participants gave to the trials used to examine memory for binding. The table shows that both for probes in which the object was old but the color had changed to another color in the array, and for intact objects in the correct color, younger children produced fewer correct responses. They did so because they misclassified the objects. In support of Figure 3, the last two columns illustrate that when binding memory is calculated only with respect to items correctly classified as old, there is still a developmental progression in working memory for binding but not in long-term memory for binding, for both re-paired and intact probes. 

#### 3.3.3. Same-Color Response Bias Rates 

Binding error rates were calculated as an average of incorrect-binding responses in both *Same Object, Different Color* and *Same Object, Same Color* trials. This means that if bias rates would differ between participants and age groups regarding the preference for one of these two response options, it should not artificially boost or lower these estimates, since a propensity to say that a color change had occurred will be correct in one trial type, but incorrect in the other. 

However, we also explored potential age differences in the tendency to indicate a color change using the Novel Object trials, in which the participants guessed that the object was studied. Because these objects were not actually studied by the participants, they allow us to explore the relative rates of guessing that the object–color binding was intact (*Same Object, Same Color)* vs. guessing that there had been a color change (*Same Object, Different Color*). In the WM trials, across all age groups, we found a tendency towards responding that the color had not changed. The younger children guessed “*Same Object, Same Color*” in an average of 67.6% of such trials, the middle children in 72.0% of such trials, and the adults in 72.6% of such trials. We found statistical evidence against age differences in these guess rates (BF_01_ = 15.53). In the LTM trials, a similar preference for the ‘*Same Object, Same Color*’ option emerged. The younger children guessed ‘same color’ in an average of 72.4% of trials, the middle children in 73.8% of trials, and the adults in 72.0% of such trials, and statistically, there was evidence against age differences in these guess rates (BF_01_ = 11.70). Overall, it appears unlikely that the age effects seen in memory binding performance can be explained by age differences in response biases. 

### 3.4. WM and LTM Correlations

Finally, we explored correlations between participants’ average WM capacity (*k*) and LTM capacity. LTM capacity was quantified as the average number of items held in LTM and estimated using *p*(LTM) × the number of items in the original array. For each participant, we computed their average WM and LTM capacity across the three different set sizes. Then, to remove potentially confounding age effects (i.e., adults might perform better than children in both the WM and LTM measures), we standardized the WM and LTM capacity scores within each age group. We observed a positive correlation between average age-standardized *k*-scores and LTM capacity scores (*r* = .44, BF_10_ = 5.78 × 10^4^), suggesting that the participants who were able to keep comparatively more items in mind than their age-matched peers in the WM task also tended to recall more items in the subsequent LTM test. 

### 3.5. Distraction Task Performance

Accuracy on the visuospatial distraction task suggested good overall engagement with this task: the youngest group (*M* = 95.0%), second-youngest (*M* = 99.0%), and adults (*M* = 100.0%).

## 4. Discussion

### 4.1. WM Capacity Limitations Constrain LTM Item Memory 

In the present study, we explored how WM capacity limitations constrain LTM in children and adults. Our results replicated previous findings, suggesting that having to remember more items in a WM task limits the subsequent LTM retrieval of items in both children and adults (Forsberg et al. 2022a). Crucially, the present study replicated these findings using the sequential presentation of items (c.f. simultaneous presentation in the original study), which indicates that this relationship is driven by WM capacity limitations, rather than by a shared perceptual encoding bottleneck. We also found that participants with a higher WM capacity remembered more items in the subsequent LTM test (similar to Forsberg et al. 2022a; see also Fukuda and Vogel 2019). Importantly, for these analyses, we standardized WM and LTM performance within each age group to rule out age confounds in both measures. The more productive LTM encoding in higher-capacity participants might partly explain the well-established correlational link between WM capacity and educational attainment (e.g., Cowan 2016; Gathercole et al. 2004) and appears aligned with suggestions that verbal short-term memory ability may predict LTM word learning in children (e.g., see Gathercole et al. 1997). 

### 4.2. The Transfer of Information between WM and LTM 

We also tested whether the ratio of information that was transferred to LTM—out of the items an individual participant could hold in WM—differed between children and adults. Similar to Forsberg et al. (2022a), we found age differences in this ratio at set size 2, such that older participants appeared to transfer these items more efficiently into LTM than younger participants. The extra boost at this set size in older participants might reflect that they tended to have extra resources available (as two items is likely below WM capacity limits in adults), perhaps to carry out deeper encoding of the two items (e.g., Craik and Lockhart 1972). However, at the larger set sizes (3 and 4 items), the ratio appeared consistent between the age groups (see Figure 2, Panel C). 

### 4.3. Memory Binding 

The ability to correctly remember WM memory bindings (i.e., item–color bindings)—when excluding instances in which the object was completely forgotten—was better at lower set sizes and in older participants. This fits with the previous literature which suggested that young children exhibit binding memory deficits compared to older children and adults (e.g., Cowan et al. 2006a, 2006b; Darling et al. 2014; Wang et al. 2015). Our results also fit with evidence suggesting that developmental increases in visual WM capacity are driven by an increase in both the number of objects that can be stored and the number of features (Forsberg et al. 2022d).

Interestingly, we found no age differences in LTM performance for bound information. While younger children were much more likely to forget the object, for the objects they did remember, they stayed relatively intact on color in LTM. In other words, in LTM, the young children forgot a great number of the objects per se, whereas older individuals only forgot the color. Thus, although children and adults remembered the binding in LTM for similar proportions of the objects that they remembered (Figure 3), there were fewer remembered objects and therefore fewer remembered bindings in younger children.

The above findings can be considered in light of fuzzy trace theory applied to child development (Brainerd and Reyna 2015). In that theoretical framework, though gist information is slow to develop, verbatim information develops more quickly. In our procedure, the use of gist information should help to retain the objects by taking into consideration their meanings. The use of verbatim information should be able to help retain objects in the short term based on how they look, along with their colors. In the WM procedure, younger children remember less of everything. One explanation for this might be that, with poorer gist representations, more time during encoding must be taken up encoding each object, leaving time for fewer objects and fewer object–color bindings. In the LTM procedure, however, younger children remember fewer objects, but not fewer object–color bindings as a proportion of the objects remembered. This might signal that the younger children have encoded fewer items (because of poorer gist representations) but have encoded them verbatim, which often affords the object–color binding for those items that are adequately encoded in LTM. This result is in keeping also with the hypothesis (Cowan 2022; Cowan et al. 2018) that, with development, there is a growth in the ability to form better patterns at the time of WM encoding, which may result in longer-lasting memories. 

One difficult-to-explain aspect of the results is why the set size effect in WM did not carry over into LTM, a finding also obtained by Bartsch et al. (2019). One way to explain this is according to a version of the embedded processes theory of memory (e.g., Cowan 1988, 1999, 2001, 2019). According to that view, a portion of LTM is activated by the stimuli in a manner that is not capacity-limited, though it is affected by time, interference, and retrieval cues. Within this activated portion of LTM, the focus of attention maintains a very limited number of items and bindings. It would be expected that the activated portion of LTM could be encoded for multiple items at once in the array, and that some of this activated information may result in permanent LTM encoding. However, information in the focus of attention regarding arbitrary bindings may be difficult to maintain in a retrievable manner in the long term, when these bindings involve the massive re-use of features (the reuse of similar colors from trial to trial). In that case, the set size effect in WM would occur because of the contribution of the focus of attention, whereas the residual LTM encoding would lose most of this privileged information and would be based primarily on activated LTM. This interpretation helps to constrain the embedded processes approach and is in keeping with a recent study (Bartsch and Oberauer 2023), indicating that the focus of attention and LTM both contribute to WM for bindings in different ways (e.g., in their abstract, the statement that “at higher set sizes performance was more stable than expected from a capacity limited memory”).

### 4.4. Implications and General Discussion 

Overall, our results are generally consistent with the theories of WM and LTM as linked, closely related systems (Cowan 2019) and suggestions that WM may act as a bottleneck for LTM learning (e.g., Forsberg et al. 2021b, 2022a; Fukuda and Vogel 2019). The strong effects of WM encoding load on subsequent LTM retrieval in our study may help explain inconsistencies in young children’s general memory abilities (Miller 2014) as they may be able to encode information quite well when WM is not overloaded, but struggle when the demands of the memory situation exceed their WM capacity. Moreover, children of the same age differ in their WM capacity, and it is estimated that around 10% of children exhibit considerable impairments (Alloway et al. 2009). Previous research has linked these deficits to difficulties in the ability to follow instructions (Jaroslawska et al. 2016) and lower educational attainment (Cowan 2022). Our findings suggest that children with poorer WM capacity, compared to their age-matched peers, also remembered less information in the LTM test.

Combined, these findings have potential implications for understanding the optimal methods of the presentation of educational material. In the classroom, the same WM load will be less overwhelming for some children than others. While likely challenging to implement in practice, our study adds to a body of evidence suggesting that adjusting the informational load to each student’s optimal load can help support efficient learning. For example, allowing learners to pause and segment recorded presentations may help reduce informational overload, which may enhance learning (Mayer 2005). Lusk et al. (2009) found that when such segmentation was allowed, learners with a lower working memory capacity were able to recall information equally well as learners with a higher capacity. However, just making sure that the load does not exceed capacity might be too simplistic. For example, Cowan et al. (2021) found that children and adults performed better in a secondary task when the memory load was lower. However, 7-year-olds performed most poorly on this secondary task at the lowest memory load of one item, perhaps reflecting boredom and zoning out in this condition. Either way, our results suggest that attempts to adjust information to prevent WM overload are likely to support better long-term learning. 

To conclude, our study adds to a growing body of research suggesting that WM overload constrains LTM learning in children as well as adults. By replicating these findings using sequential presentation, the study indicates that WM capacity limitations—rather than a perceptual bottleneck—drive these constraints. Further work is needed to explore this relationship for memory bindings of complex, educationally relevant materials. 

## Figures and Tables

**Table 1:** Averages for transformed data by set size and age group.

Set Size	*p*(WM) Item	*P*(LTM) Item	LTM/WM Item Ratio	WM Binding Errors	LTM Binding Errors	WM Capacity (*k*)
1st and 2nd graders						
Two items	.79 (.13)	.30 (.15)	0.37 (0.18)	.31 (.12)	.43 (.24)	1.6 (0.26)
Three items	.69 (.20)	.29 (.16)	0.56 (0.30)	.38 (.11)	.47 (.17)	2.1 (0.59)
Four items	.67 (.19)	.27 (.17)	0.43 (0.28)	.43 (.15)	.48 (.23)	2.7 (0.77)
5th through 7th graders						
Two items	.93 (.06)	.53 (.19)	0.57 (0.21)	.18 (.10)	.44 (.17)	1.9 (0.12)
Three items	.89 (.11)	.47 (.21)	0.53 (0.23)	.33 (.13)	.46 (.17)	2.7 (0.32)
Four items	.83 (.13)	.41 (.23)	0.50 (0.27)	.40 (.14)	.42 (.17)	3.3 (0.52)
Adults						
Two items	.95 (.06)	.52 (.20)	0.55 (0.21)	.14 (.09)	.43 (.18)	1.9 (0.11)
Three items	.93 (.07)	.50 (.21)	0.54 (0.21)	.36 (.13)	.44 (.18)	2.8 (0.22)
Four items	.90 (.09)	.47 (.19)	0.51 (0.18)	.35 (.10)	.47 (.16)	3.6 (0.37)

Note. Values in parenthesis represent standard deviations.

**Table 2:** Frequencies of each response type (out of 20 possible in WM and 24 possible in LTM) for trials used to examine binding memory and binding estimates.

**Trials with Old Item, Different Color from Array**								
	**Working Memory Trials**			**Long-term Memory Trials**				
	“Old”	“DiffCol”	DiffObj”	“Old”	“DiffCol”	“DiffObj”	WMb	LTMb
**Youngest Children**								
Mean	6.98	**8.60**	4.42	2.77	**4.49**	16.74	0.55	0.57
SD	3.14	3.05	2.59	2.36	3.67	4.55	0.19	0.30
**Middle Children**								
Mean	6.44	**11.41**	2.15	5.36	**7.56**	11.08	0.64	0.60
SD	2.64	2.77	1.35	3.17	2.93	4.09	0.15	0.20
**Adults**								
Mean	5.62	**13.07**	1.31	5.62	**9.50**	8.88	0.70	0.63
SD	2.98	3.27	1.16	2.53	2.91	3.86	0.16	0.13
**Trials with Item and Color Intact**								
	**Working Memory Trials**			**Long-term Memory Trials**				
	“Old”	“DiffCol”	“DiffObj”	“Old”	“DiffCol”	DiffObj”	WMb	LTMb
**Youngest Children**								
Mean	**22.19**	9.91	7.91	**3.26**	3.84	16.91	0.69	0.47
SD	6.19	4.22	5.04	2.98	3.18	4.59	0.14	0.31
**Middle Children**								
Mean	**28.33**	8.54	3.13	**7.62**	6.31	10.08	0.77	0.53
SD	4.99	3.94	2.36	4.08	2.76	5.15	0.11	0.19
**Adults**								
Mean	**30.90**	7.10	2.00	**7.29**	7.64	9.07	0.81	0.48
SD	4.39	2.92	2.34	3.44	2.82	4.06	0.08	0.15

Note. Results are cumulative across set sizes. WM = working memory tests; LTM = long-term memory tests, WMb = working memory binding, LTMb = long-term memory binding. Binding scores were calculated here by the number of correct responses divided by the number of trials in which the item was correctly identified as old, calculated separately for trials in which the object and color were re-paired (top half of table) or intact from the array (bottom half of table). Two of the youngest children had no correctly identified objects in LTM upon which the binding could be calculated; one child for changed objects and the other child for either changed or intact objects. Participant responses: “Old” = old object intact, “DiffCol” = old object with wrong color, “DiffObj” = new object. Bolded cells indicate mean frequencies for the correct answers.

## Data Availability

The original data and analysis code is available online at https://osf.io/9zvrm/.

## Note

1 We use the term visual WM to describe our procedure, but note that some might find term ‘visual short-term memory’ more appropriate, and some consider the two terms interchangeable (Cowan 2017).
